# Surveillance of Community Outbreaks of Respiratory Tract Infections Based on House-Call Visits in the Metropolitan Area of Athens, Greece

**DOI:** 10.1371/journal.pone.0040310

**Published:** 2012-08-08

**Authors:** Alex Spanos, George Theocharis, Drosos E. Karageorgopoulos, George Peppas, Dimitris Fouskakis, Matthew E. Falagas

**Affiliations:** 1 Alfa Institute of Biomedical Sciences (AIBS), Marousi, Athens, Greece; 2 SOS Doctors, Athens, Greece; 3 Department of Mathematics, National Technical University of Athens, Athens, Greece; 4 Department of Medicine, Henry Dunant Hospital, Athens, Greece; 5 Department of Medicine, Tufts University School of Medicine, Boston, Massachusetts, United States of America; University of Calgary & ProvLab Alberta, Canada

## Abstract

**Background:**

The traditional Serfling-type approach for influenza-like illness surveillance requires long historical time-series. We retrospectively evaluated the use of recent, short, historical time-series for recognizing the onset of community outbreaks of respiratory tract infections (RTIs).

**Methods:**

The data used referred to the proportion of diagnoses for upper or lower RTIs to total diagnoses for house-call visits, performed by a private network of medical specialists (SOS Doctors) in the metropolitan area of Athens, Greece, between January 01, 2000 and October 12, 2008. The reference standard classification of the observations was obtained by generating epidemic thresholds after analyzing the full 9-year period. We evaluated two different alert generating methods [simple regression and cumulative sum (CUSUM), respectively], under a range of input parameters, using data for the previous running 4–6 week period. These methods were applied if the previous weeks contained non-aberrant observations.

**Results:**

We found that the CUSUM model with a specific set of parameters performed marginally better than simple regression for both groups. The best results (sensitivity, specificity) for simple regression and CUSUM models for upper RTIs were (1.00, 0.82) and (0.94, 0.93) respectively. Corresponding results for lower RTIs were (1.00, 0.80) and (0.93, 0.91) respectively.

**Conclusions:**

Short-term data for house-call visits can be used rather reliably to identify respiratory tract outbreaks in the community using simple regression and CUSUM methods. Such surveillance models could be particularly useful when a large historical database is either unavailable or inaccurate and, thus, traditional methods are not optimal.

## Introduction

Various genera of respiratory viruses, including rhinovirus, parainfluenza virus, adenovirus, respiratory syncytial virus, human metapneumovirus, coronavirus, and, particularly, influenza virus circulate each year in the community [1], [2]. They are associated with upper and lower respiratory tract infections (RTIs), from the common cold to viral pneumonia, in children and adults. Community outbreaks of RTIs can be associated with considerable morbidity and mortality either due to the infection *per se* or due to the exacerbation of other diseases, such as those that affect the cardiovascular and respiratory system [3].

The development of new methods for early recognition or prediction of community outbreaks or the improvement of the currently used methods is of increasing scientific and public health interest [4]. This process is primarily based on monitoring relevant surveillance data for potential clustering of cases of RTIs that exceed an expected threshold. Surveillance for influenza-like illness is primarily conducted through networks of sentinel providers. Cases are defined as individuals who present clinical manifestations matching specifically defined clinical criteria of the syndrome.

The analysis of surveillance data on RTIs for the purpose of identifying the occurrence of epidemics requires advanced statistical methods. A common family of such methods derives from the innovatory application of a periodic regression model on pneumonia and influenza mortality data, presented by R.E. Serfling in 1963 [5]. The general principle of this type of method is the use of long-term historical data for estimating expected values for future time-dependent upper thresholds of the relevant distribution, to which the actual observations made are subsequently compared [5], [6]. Alternative sets of statistical methods that use the same general principle, but require input of relatively few recent historical data have also been proposed, yielding favorable results [7], [8]. Such statistical methods belong to the broader classes of time-series, regression and industrial quality control methods. Cumulative sum (CUSUM) statistics of the latter class, are nowadays widely used in biomedical research [7], [8], [9] and constitute one of the components of the Early Aberration and Reporting System (EARS) that is used for syndromic surveillance by the United States Centers for Disease Control and Prevention [9].

We sought to establish optimal models for recognition of the onset of respiratory tract infection outbreaks in the metropolitan area of Athens, Greece, on the basis of short-term data from house-call visits, by applying either simple regression or CUSUM methods [7]. These methods, apart from their ability to function on the basis of short-term data, also share the benefit of relatively simple implementation.

## Methods

### Data sources

We used historical data from the computerized database of the SOS Doctors private network of medical specialists. This network performs emergency, fee-per-service, house-call visits for both adults and children on a 24-hour basis at the metropolitan area of Athens, Attica, Greece, that is populated by approximately 5 million people. The data we used referred to the 9-year period between January 01, 2000 and December 10, 2008 and were recorded by a total of 38 SOS Doctors specialists. Briefly, on each house visit they filled a specially designed form with data for the chief complaint of the patient, present illness, past medical and surgical history, physical examination, overall clinical assessment, likely diagnosis and management plan. They also filed the primary diagnosis into prespecified diagnostic categories (including upper or lower RTIs). As a general rule, lower RTIs were considered those affecting the respiratory tract below the level of the vocal cords. The operational characteristics of SOS Doctors including the methodology used for the collection and archiving of clinical data as well as the main characteristics of the patient population they serve have been described previously in detail [10]. Subsets of the database we used have also been analyzed in previous studies assessing the effects of meteorological variables on respiratory and lower urinary tract infections [11], [12].

The primary diagnosis for each house-call visit was registered by the physician who visited and examined the patient in a specifically designed report form, along with other relevant information, and was promptly entered in the computerized database of SOS Doctors by secretarial staff. We analyzed visits for upper and lower RTIs separately.

The type of observations chosen for our analysis is the weekly-averaged proportion of house-call visits for upper (or lower) RTIs to total house-call visits. Thus, observation *y_t_* is the weekly-averaged proportion of upper (or lower) RTIs to the total house visits during week *t*.

### Reference standard for classification of observations

To obtain an *a priori* classification of the observations as normal (non-epidemic) or aberrant (epidemic), i.e., a reference standard with which the results of the application of our models could be compared, we used the application developed by Pelat et al. [13]. This freeware online application can apply periodic regression models on user-uploaded data (related to infection incidence) so that baseline levels and “*epidemic thresholds*” can be estimated, either retrospectively or prospectively. When conducting retrospective analysis, the application proceeds to classify each observation as normal (within the epidemic threshold) or aberrant (exceeding the epidemic threshold).

The above described application provides the option for various user-defined settings. To estimate the reference baseline levels and epidemic thresholds for our data sets (upper and lower RTIs), a periodic regression model with cubic trend and annual periodicity was used. Trend was chosen as cubic, as we sought to obtain the best possible fit, and annual periodicity was chosen with respect to the expected seasonality for most common RTIs [14], as previously observed for our data set [12]. To estimate baseline levels for our data set we removed the highest 20% percentile of the observations, which we considered to represent aberrant values. Finally, we used an upper 97.5% one-sided confidence interval around the calculated baseline levels to define the epidemic thresholds.

### Models for recognition of outbreaks using short-term data

We applied two types of statistical methods for the recognition of the onset of community outbreaks of respiratory tract infections (simple regression and CUSUM, respectively) requiring historical data for the previous 4–6 weeks. These alert-generating methods decide whether to create or not an “*alert*” for each week *t*. The decision rule for creating an alert depends on the comparison of the value of a model-specific variable and a corresponding threshold. This comparison has two possible outcomes: if the threshold is exceeded, the observation is flagged as an alert; otherwise it is not flagged and is labeled as “expected”.

#### Simple regression

Using the simple regression model [7], the threshold for each week *t* is defined as the upper limit of the Student 100(1−*a*)% confidence interval for the unknown mean (*degrees of freedom = m−1*):


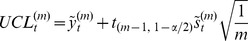


In the above formula 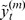 and 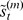 symbolize the *m*-week running means and running standard deviations, respectively, calculated from weeks *t−m,…,t−1*, while 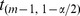 is the appropriate t-statistic for *m−1* degrees of freedom and significance level *α*. The assumption of the method is that each running set of *m* observations belongs to a normal distribution. For the parameter *m*, that represents the number of weeks analyzed prior to the week *t*, we assigned values between 4–6. We set the significance level *α* for the confidence interval to 0.10.

#### CUSUMs

CUSUMs are methods that are used to detect small aberrations from an *in-control* process [15], [16], [17]. The *m*-week standardized upper CUSUM is calculated as:


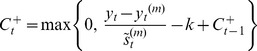


In this formula *k* stands for the standardized *reference value K* of the CUSUM; it is measured in standard deviations, and represents the maximum allowed range of fluctuation around the process mean for the in-control process. *K* is usually defined as the intermediate value between the in-control process mean and the out-of-control process *target* mean. In the context of this analysis, the in-control process is represented by the normal observations (non-epidemic), while the out-of-control process is represented by the epidemic observations. An alarm is raised by the method if the value of the CUSUM exceeds a pre-specified value *h*. The parameter *h*, measured in standard deviations, is referred to as the standardized *decision value* for the CUSUM and represents the threshold of the method.

The selection of appropriate values for the above-mentioned parameters *(k, h)* for a CUSUM procedure takes into account the in-control (average run length) *ARL* of the in-control process [18], [19], [20]. The *ARL* is a measure of the time interval between false alarms, therefore it is analogous to a Type I error. In order to understand the comparability of *(k, h)* parameters it should be mentioned that for a fixed *k* value, increasing the *h* value would generally result in lower sensitivity and higher specificity results (and vice-versa). The same applies for *k*, for a fixed *h* value [15]. For the purposes of this study we tested two sets of values: a) (*k, h*) = (0.5, 5), which is a commonly used set for a CUSUM procedure [18], [19], [20], [21], and b) (*k, h*) = (0.75, 1.53). The first set corresponds to an *ARL* of 930 weeks. For the second set of values, *k* was selected for the CUSUM to detect shifts of *Δ* = 2*k* = 1.5*σ*, and *h* was calculated with respect to a specific *ARL* approximately representing the desired Type I error rate *α = 0.10* (as in the simple regression method). Hence, this *ARL* was selected to be 45, roughly one tenth of the total 467 observations. The *h* value, given *k* and *ARL*, was computed as 1.53. As in the simple regression model described above, we assigned values for *m* between 4–6 (weeks).

### Details for the application of the methods

The objective in this study is to assess the performance of the methods in identifying the onset of RTI outbreaks. We considered that the onset of an outbreak corresponds to the first aberrant value according to the reference standard classification. In this regard, we applied the simple regression and CUSUM models for detection of an outbreak at week t, only if the observations for the prior *m*-week interval were all classified as non-aberrant by the reference standard classification. This rule we applied satisfies the simple regression and CUSUM assumptions for normally distributed observations and in-control processes, respectively. By taking this action, an unavoidable consequence is the reduction of the total amount of runs for the methods.

### Evaluation of performance of models

The alert generating methods of simple regression and CUSUMs were applied on our retrospective time-series data set. Applying each of the models for every week *t* assessed, we obtained an output as either alert or non-alert. We compared this output to the classification of the same observation as normal or aberration according to the reference standard method. We then constructed for each of the models a 2×2 table listing the true/false positive and true/false negative observations. From these tables, we calculated point estimates and confidence intervals (calculated with the Wilson score method for sensitivity, specificity for each model and specific set of model parameters selected [22]. Analyses took place in MATLAB (2010a, The Mathworks Inc., Natick MA, 2010) and R [23].

## Results

**Figure 1:**
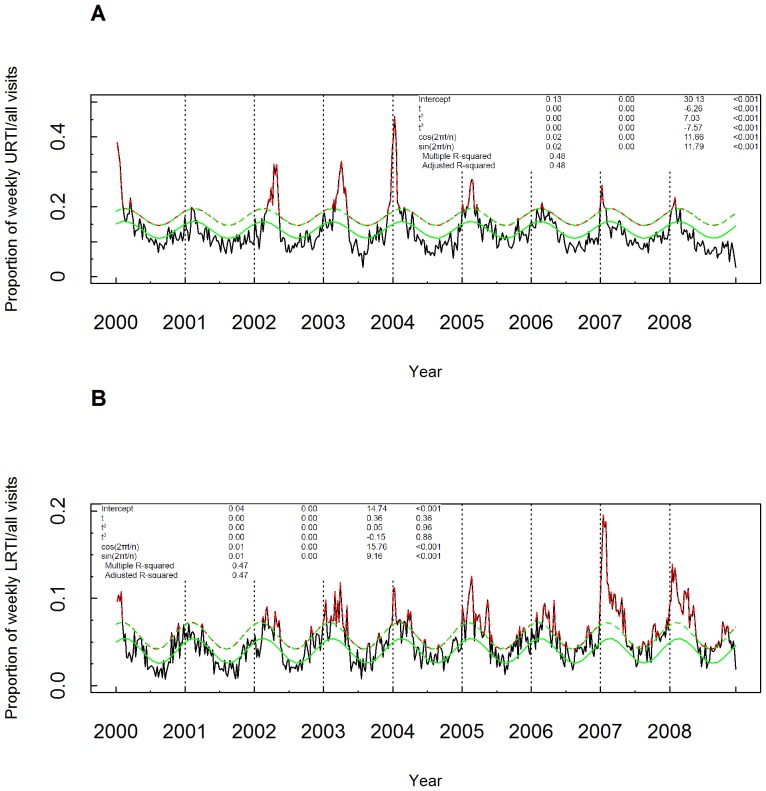
Sequence plots of time-series of observations of weekly-averaged proportions of A) upper and B) lower respiratory tract infections to total house visits in the area of Athens, Greece as recorded by SOS Doctors from January 01, 2000 to October 12, 2008. Superimposed are the results of the reference standard classification procedure. The green continuous line represents estimated baseline level. Green broken line over baseline represents threshold. The observations identified as unexpected have been plotted in red color.

The analysis was performed on 467 observations of weekly-adjusted average proportions of upper or lower RTI diagnoses for house call visits, between January 01, 2000 and October 12, 2008; the average (minimum-maximum) respective values were 0.13 (0.03–0.46), and 0.05 (0.01, 0.20). For upper and lower RTIs, 83/467 (17.8%) and 74/467 (15.8%) of the respective observations were classified as aberrations by the reference standard procedure. Despite selecting periodic regression model fittings that included a trend component of up to cubic order, the best fit model for upper and lower RTIs solely included a constant trend component. In Figures 1a and 1b, we provide sequence plots for both groups of observations (upper and lower RTIs, respectively), superimposing the reference standard classification, accompanied by the results of the statistical model fitting.

In Tables 1 and 2, we present the summary findings for sensitivity, specificity of simple regression and CUSUM models for the detection of upper and lower RTI outbreaks, respectively, with regard to each set of input parameters used, along with the respective 95% confidence intervals. The total amount of comparisons executed by the models for the calculation of these indices is also shown.

**Table 1:** Method performance for upper respiratory tract infections.

Methods	Parameter combinations	Sensitivity	95% CI for sensitivity	Specificity	95% CI for specificity	Observations used to total observations (%)*
Simple Regression (α = 0.10)	m = 4	1.00	(0.99–1.00)	0.82	(0.77–0.86)	305/463 (65.9%)
	m = 5	1.00	(0.99–1.00)	0.79	(0.74–0.83)	284/462 (61.5%)
	m = 6	1.00	(0.99–1.00)	0.77	(0.72–0.82)	266/461 (57.7%)
CUSUM	(k,h) = (0.5, 5)					
	m = 4	0.50	(0.44–0.56)	0.98	(0.95–0.99)	305/463 (65.9%)
	m = 5	0.59	(0.53–0.64)	0.98	(0.96–0.99)	284/462 (61.5%)
	m = 6	0.53	(0.47–0.59)	0.99	(0.97–1.00)	266/461 (57.7%)
	(k,h) = (0.75, 1.53)					
	m = 4	0.85	(0.61– 0.96)	0.91	(0.87–0.94)	305/463 (65.9%)
	m = 5	0.94	(0.69–1.00)	0.93	(0.88–0.95)	284/462 (61.5%)
	m = 6	0.87	(0.58–0.98)	0.94	(0.90–0.96)	266/461 (57.7%)

* Only prior observations referring to a non-epidemic period were used (see text).

**Table 2:** Method performance for lower respiratory tract infections.

Methods	Parameter combinations	Sensitivity	95% CI for sensitivity	Specificity	95% CI for specificity	Observations used to total observations (%)*
Simple Regression (*α* = 0.10)	m = 4	1.00	(0.99–1.00)	0.80	(0.76–0.84)	316/463 (68.3%)
	m = 5	1.00	(0.99–1.00)	0.76	(0.70–0.80)	298/462 (64.5%)
	m = 6	1.00	(0.99–1.00)	0.73	(0.68–0.78)	282/461 (61.2%)
CUSUM	(k,h) = (0.5, 5)					
	m = 4	0.35	(0.30–0.40)	0.97	(0.94–0.98)	316/463 (68.3%)
	m = 5	0.47	(0.41–0.52)	0.98	(0.96–0.99)	298/462 (64.5%)
	m = 6	0.43	(0.37–0.49)	0.97	(0.95–0.99)	282/461 (61.2%)
	(k,h) = (0.75, 1.53)					
	m = 4	0.94	(0.69–1.00)	0.90	(0.86–0.93)	316/463 (68.3%)
	m = 5	0.93	(0.66–1.00)	0.91	(0.87–0.94)	298/462 (64.5%)
	m = 6	0.86	(0.56–0.97)	0.90	(0.86–0.93)	282/461 (61.2%)

* Only prior observations referring to a non-epidemic period were used (see text).

The performance of the simple regression and CUSUM models under the different set of parameters we selected, generally yielded high values for sensitivity and specificity for both types of RTIs. The main exception was observed when the CUSUM model was run with the “typical” set of parameters for *(k, h)* of (0.5, 5), which yielded high values for specificity but low values for sensitivity.

Simple regression displayed excellent sensitivity results for both groups of RTIs. As the *m*-weeks parameter increased, simple regression displayed slightly lower specificity for both groups of RTIs. The best results were obtained with the parameter selection for *m* = 4 weeks. Simple regression seemed to perform somewhat better for the upper RTIs compared to the lower RTIs.

Concerning the use of CUSUM models, the typical (*k, h*) = (0.5, 5) set of parameters yielded high values for specificity but low values for sensitivity for both upper and lower RTIs. However, the (*k, h*) = (0.75, 1.53) set achieved values for sensitivity exceeding 0.85 (upper RTIs) and 0.86 (lower RTIs) and values of specificity above 0.91 (upper RTIs) and 0.90 (lower RTIs). Marginally best results were obtained for the parameter selection of *m* = 5 weeks. As with simple regression, CUSUM methods altogether seemed to perform somewhat better for the upper RTIs compared to the lower RTIs. By comparing the simple regression and the CUSUM models on the basis of performance, the CUSUM model with *m* = 5 and (*k*, *h*) = (0.75, 1.53) was superior for both upper and lower RTIs. The methods computed thresholds and executed comparisons only for the subset of observations, for which the previous m-week intervals contained zero aberrant observations (as classified by the reference standard procedure). Including this rule omitted a total number of 158, 178, 195 (34.1%, 38.5%, 42.3%) possible method “runs” for upper RTIs and 147,164, 179 (31.7%, 35.5%, 38.8%) for lower RTIs.

## Discussion

The application of two different models (simple regression and CUSUM models, respectively) for early detection of community outbreaks of upper or lower RTIs using short-term historical data of house-call visits provided favorable results in terms of sensitivity and specificity, compared with a reference standard procedure based on long-term relevant historical data. In particular, a CUSUM model using a specific set of parameters optimized for our dataset seemed to be the best-fit one.

Such methods as those we evaluated could be implemented where extensive databases tracking the incidence of infectious syndromes are unavailable or unreliable. This is frequently the case in developing countries. As exemplified by the recent H1N1 pandemic, epidemics can emerge in areas with a relatively low level of public health surveillance [24]. The simple regression and CUSUM models we evaluated in this study appear to perform reasonably well in detecting the onset of an outbreak at week *t*, with the only prerequisite being the availability of prior relevant data for a non-epidemic period of only 4–6 weeks duration to be used as the comparator. The selection of the 4–6 week running period was rather arbitrary. We believe that the comparison of the index week *t* with a shortest period would lead to non-significant findings; a longer period might also include data from prior outbreaks which would also be inappropriate for the comparison.

Data for house-call visits performed by a network of medical specialists affiliated to a private company, such as SOS Doctors in Athens, Greece, can have several advantages for use for syndromic surveillance purposes [10], [25]. The data we evaluated are ordinarily collected in a real-time manner by the private company for own purposes. Therefore, no additional costs are entailed for the collection of this type of data. House-call visits refer directly to the community, where outbreaks of viral infections first occur. Visits are done on an emergency basis, when the patient feels mostly in need for a physician, and, supposedly, clinical manifestations are more obvious. In contrast, disease surveillance data collected by visits to sentinel providers may selectively refer to patients that can go by themselves or be transferred to the ambulatory office of the providers at a time that this is feasible [25].

The most important bias in the synthesis of the patient population served by SOS Doctors and other relevant private networks is the financial ability of the patients to pay for the visit. This could be particularly important during financial crises, as it may limit the representativeness of the sample with regard to the total population. Another issue is the accessibility of the patients to other types of emergency healthcare services, which can be limited for patients with physical or mental disabilities. Depending on the operational activity of the private network of physicians, the data for house call visits can refer to a large number of patients. Minor deviations from the epidemic threshold can then be marked by statistical significance, leading to prompt detection of outbreaks and timely effectuation of the appropriate public health measures [26], [27]. Although different segments of the population can be affected at a different extent or severity during specific community viral outbreaks, none of the segments is, most commonly, spared [28], [29].

Our analysis is subject to many potential limitations. At the data level, clinical differentiation between upper and lower RTIs is not always evident. Individual physicians in the network of SOS Doctors may have used a different relevant diagnostic approach. The use of a standardized case-definition for syndromic surveillance could have helped in this regard [30]. We analyzed both upper and lower RTIs, so that our findings would not be affected from bias by potential misclassifications. Still, the upper RTIs might more clearly be associated with community outbreak of viral illnesses than lower RTIs. Accordingly, the models we used for short-term recognition of community outbreaks of respiratory tract infection performed better for upper RTIs. Instead of daily values, we selected to analyze weekly-averaged values for RTIs to minimize the likelihood for high auto-correlation or variability between successive observations. We also chose to analyze the proportion of the visits for upper or lower RTIs to the total house call visits, instead of the absolute counts of RTIs, to minimize the effect of various factors, such as seasonal variation in morbidity, meteorological conditions, vacation periods, or strikes in the public healthcare sector, which could influence the demand for private house-call visits [3], [10].

At the statistical modeling level, potential limitations involve the unavoidably arbitrary, to some extent, selection of model parameters. Results for the types of statistical analysis we performed have absolute dependence on parameter input. Parameters need to be robustly calibrated in order to achieve a desired balance in the trade-off between sensitivity and specificity, keeping in mind the consequences of false alarms and the benefits of true alarms [20]. Unfortunately, optimal calibrations prove to be database-dependent. This concept applies to both the reference standard, and to the alert-generating methods application procedures. For example, decrease in specificity for the alert-generating methods we evaluated might have resulted from earlier identification of an outbreak than the reference standard method.

Particularly for the CUSUM methods, global standard values for (*k*, *h*) cannot be determined, as they are dependent to the process' in control average run length, which is in turn dependent on observation distribution. This could also constitute a shortcoming of the CUSUM methodology compared to the substantially simpler in principle simple regression methodology when executing prospective analysis. Determination of the best-fit CUSUM model for a specific data set would require the evaluation of long-term historical data which might not be available.

Finally, we did not evaluate the simple regression and CUSUM models prospectively. We analyzed a large data-set of historical data as the reference standard for characterization of the outbreaks. Future studies aiming to prospectively evaluate data from house call visits for the detection of outbreaks of respiratory infections could use data from official sentinel surveillance networks, if available, as the reference standard [25]. We can only assume the performance of the models used for the detection of outbreaks after the outbreak has begun. The models might continue generating an alert for the phase of the outbreak that the number of cases steadily increases, whereas alert generation might cease once the outbreak peaks and in the phase that the number of cases decline.

Sophisticated time-series (hierarchical models), Bayesian inference (hidden Markov models) and other types of methods [31], [32], [33], [34], [35], [36] are increasingly gaining popularity in the field of statistical outbreak detection, providing a framework for future research in the field. A broad review of the contextual application of these methods has been conducted by Unkel et al. [37].

In conclusion, electronic health records for house call visits might serve as an alternative surveillance network for the prediction of respiratory tract outbreaks in the community using short-term data (for the previous 4–6 weeks). Both methods we evaluated (simple regression and CUSUM, respectively) performed reasonably well, in this regard, showing excellent sensitivity and very good specificity, when compared to a reference standard classification that used long-term historical data. A CUSUM model with a specific set of parameters appeared to be the best-fit one. The determination of the optimal set of parameters may, however, be database-specific, and it could require the evaluation of long historical time-series. Simple regression models could be elected if long-term historical data are lacking. Additional studies are needed in order to validate the performance of the above models in the prospective detection of the onset of RTI outbreaks on the basis of data for house call visits.
